# Identification of low molecular weight nuclear complexes containing integrase during the early stages of HIV-1 infection

**DOI:** 10.1186/1742-4690-10-13

**Published:** 2013-02-01

**Authors:** Annabelle Gérard, Nicolas Soler, Emmanuel Ségéral, Michael Belshan, Stéphane Emiliani

**Affiliations:** 1 Inserm, U1016, Institut Cochin, Paris, France; 2 Cnrs, UMR8104, Paris, France; 3 Université Paris Descartes, Paris, France; 4 Department of Medical Microbiology and Immunology, Creighton University School of Medicine, Omaha, NE, USA; 5 The Nebraska Center for Virology, Lincoln, NE, USA

**Keywords:** Human immunodeficiency virus, Integrase, Pre-integration complex, LEDGF/p75

## Abstract

**Background:**

HIV-1 replication requires integration of its reverse transcribed viral cDNA into a host cell chromosome. The DNA cutting and joining reactions associated to this key step are catalyzed by the viral protein integrase (IN). In infected cells, IN binds the viral cDNA, together with viral and cellular proteins, to form large nucleoprotein complexes. However, the dynamics of IN complexes formation is still poorly understood.

**Results:**

Here, we characterized IN complexes during the early stages of T-lymphocyte infection. We found that following viral entry into the host cell, IN was rapidly targeted to proteasome-mediated degradation. Interactions between IN and cellular cofactors LEDGF/p75 and TNPO3 were detected as early as 6 h post-infection. Size exclusion chromatography of infected cell extracts revealed distinct IN complexes *in vivo*. While at 2 h post-infection the majority of IN eluted within a high molecular weight complex competent for integration (IN complex I), IN was also detected in a low molecular weight complex devoid of full-length viral cDNA (IN complex II, ~440 KDa). At 6 h post-infection the relative proportion of IN complex II increased. Inhibition of reverse transcription or integration did not alter the elution profile of IN complex II in infected cells. However, in cells depleted for LEDGF/p75 IN complex II shifted to a lower molecular weight complex (IN complex III, ~150 KDa) containing multimers of IN. Notably, cell fractionation experiments indicated that both IN complex II and III were exclusively nuclear. Finally, IN complex II was not detected in cells infected with a virus harboring a mutated IN defective for LEDGF/p75 interaction and tetramerization.

**Conclusions:**

Our findings indicate that, shortly after viral entry, a significant portion of DNA–free IN that is distinct from active pre-integration complexes accumulates in the nucleus.

## Background

During the early stages of retroviral replication, the virus travels from the cellular plasma membrane across the nuclear pore to finally integrate its viral cDNA into the host cell genome. These early events first require the reverse transcription of the viral RNA into a linear double strand cDNA copy by the viral reverse transcriptase (RT). Once synthesized, this cDNA becomes part of a large nucleoprotein complex, called the pre-integration complex (PIC) (reviewed in [1]). PICs from Moloney murine leukemia virus (MLV) [2-4] or Human immunodeficiency virus (HIV) [5-7] can be partially purified after cell infection and can efficiently integrate their associated reverse transcribed viral cDNA into heterologous DNA targets *in vitro*[8]. The integration reaction is mediated by the retroviral integrase (IN) [9-11]. Within the PIC, IN binds to viral cDNA ends [12-14] and catalyzes the DNA cutting and joining reactions. First, the 3′ processing reaction consists in the hydrolysis of a dinucleotide at each end of the viral cDNA [4,15,16]. Then, exposed recessed 3′ hydroxyl groups of the viral cDNA are joined to the 5′ ends of the cut host target DNA [4-6,15]. At this stage, cellular enzymes are probably in charge of removing the 5′ unpaired viral DNA ends and subsequently catalyze the gap filling and ligation reactions of host-viral DNA junctions [17]. Human immunodeficiency virus type-1 (HIV-1) integration produces a 5 bp duplication of the DNA host sequence at each end of the integrated provirus [18].

Retroviral PICs are large nucleoprotein complexes that contain several viral and cellular proteins in addition to IN and viral cDNA. Biochemical studies indicate that HIV-1 PICs contain the viral nucleocapsid (NC), matrix (MA), Vpr and RT proteins [19-25]. In contrast with MLV [2,26], HIV-1 PICs were shown to be devoid of CA [19-25]. In addition, cellular proteins including barrier to auto-integration factor (BAF), high mobility group protein HMGA, Ku and LEDGF/p75 have been found to associate with partially purified HIV-1 PICs [20,27-29].

HIV and other lentiviruses have the ability to infect non-dividing cells, such as terminally differentiated macrophages. Therefore, this large viral nucleoprotein complex (>50 nm) must pass through the nuclear pore with the active participation of cellular factors involved in nucleo-cytoplasmic shuttling. Although several viral proteins within the HIV-1 PIC contain karyophilic signals (MA, Vpr and IN), their exact role during PIC translocation into the nucleus is still controversial [30]. The central polypurine track (cPPT), a *cis*-acting sequence that forms a short triple stranded DNA structure (the central DNA Flap) during reverse transcription, is also implicated in the nuclear import of HIV PICs [31,32]. Importantly, reports show that the HIV-1 capsid (CA) is the dominant viral determinant for HIV-1 infection of non-dividing cells, and the kinetic of dissociation of CA from the viral core appears to be a critical step in controlling nuclear import [33,34].

Among the HIV-dependency factors involved in HIV-1 replication, TNPO3 was recently shown to be involved in a nuclear import and/or preintegration step [35-40]. TNPO3 is a karyopherin from the importin-ß family that mediates transport of serine/arginine rich (SR) proteins into the nucleus in a phosphorylation-dependent manner [41]. Using yeast two-hybrid screenings, we identified TNPO3 as a binding partner of IN [36,42]. Although a direct interaction between HIV-1 IN and TNPO3 has been clearly established [36,43,44], recent reports indicated that HIV CA is one of the viral determinants important for TNPO3 requirement [8,38-40,44,45].

Once inside the nucleus, IN catalyzes viral cDNA integration into the genome of the host cell [46,47]. HIV-1 integration occurs preferentially in transcription units (TUs) of transcriptionally active genes whereas CpG islands and promoter regions are disfavored. Importantly, the targeting of viral integration to specific regions of the host chromosome is under the control of LEDGF/p75 [48-50]. LEDGF/p75 is a key factor of HIV-1 integration that was identified as an IN interacting factor [51-53]. LEDGF/p75 is a cellular chromatin-associated protein presumably involved in transcriptional regulation of cellular genes [54-56]. LEDGF/p75 is tightly associated to chromatin, and the molecular basis of this interaction involves its conserved PWWP and AT-hook domains in the N-terminal region of the protein [57,58]. LEDGF/p75 plays an important role in lentiviral cDNA integration, as demonstrated by mutagenesis [52,59-61], over-expression of LEDGF/p75 IBD (Integrase Binding Domain) [62,63] as well as RNAi and knock-out studies [49,50,62-65]. Structural studies revealed the roles of both the catalytic core domain dimeric interface and the N-terminal domain of IN for high affinity binding to IBD [60,66,67]. Albeit not strictly essential for replication, LEDGF/p75 tethers PIC-associated IN to chromatin to presumably stimulate its enzymatic activity at the site of integration [57,58].

In this study, we explored at early times post infection the dynamics of interaction between IN and its cellular and viral partners. However, the detection of IN in infected cells remains technically challenging. We took advantage of a previously characterized virus carrying an active tagged-IN with the HA epitope at the C-terminus [68] to purify and characterize IN complexes in the context of infected lymphocytes. Our results shed light on the stability and distribution of IN during early steps of HIV-1 infection. We show that IN is rapidly degraded in a proteasome-dependent manner upon virus entry into the cell. Immunoprecipitation experiments allowed us to detect interactions between IN and its cofactors LEDGF/p75 and TNPO3 at 6 h post-infection (p.i.). Using size exclusion chromatography, we uncover that IN exists in at least two distinct complexes in infected cells: a high molecular weight complex that co-fractionates with viral cDNA and integration activity, and a low molecular weight complex devoid of viral cDNA that is found exclusively in the nucleus and depends on LEDGF/p75 expression.

## Results

### HIV-1 integrase is rapidly degraded in a proteasome-dependent manner upon cell infection

**Figure 1:**
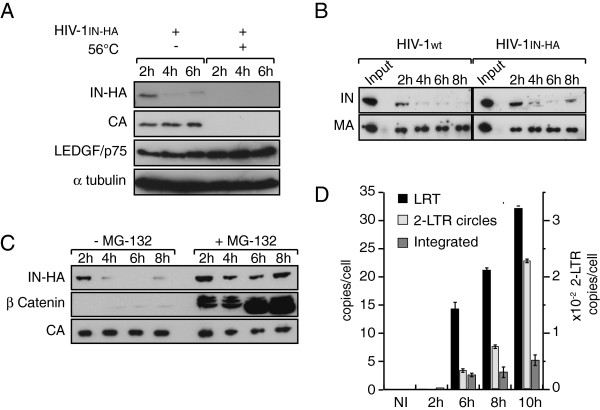
**IN is rapidly degraded in a proteasome-dependent manner during early steps of HIV-1 replication. **(**A**) SupT1 cells were infected with HIV-1_IN-HA_ or heat-inactivated virus (56°C). At indicated time post infection, cells were lysed and equivalent amounts of each sample (100 μg of protein) were analyzed using Western blotting with antibodies against HA, CA, LEDGF/p75 and α-tubulin as loading control. (**B**) Both IN-HA and non-tagged IN are rapidly degraded following viral entry into the cell. SupT1 cells were infected with HIV-1_IN-HA _or HIV-1_wt_. At indicated time post infection, cells were lysed and equivalent amounts of each sample (100 μg of protein) were analyzed using Western blotting with antibodies against IN and MA. Input represents 0.1& of the amount of virus used to infect the cells. (**C**) IN is targeted to proteasomal degradation following infection of the cells. SupT1 cells were infected with HIV-1_IN-HA _in absence or presence of proteasome inhibitor MG-132. As in (**B**), cell lysates were analyzed by Western blotting with antibodies against HA, β-Catenin and CA. β-Catenin was used as a control to monitor the efficiency of MG-132 treatment. (**D**) Proviral integrated DNA is readily detected at 6 h p.i. SupT1 cells were infected with HIV-1_IN-HA_, and DNA was extracted at indicated time post infection. Late reverse transcription product (LRT), 2-LTR circles and integrated viral DNA were quantified by real time PCR.

To facilitate HIV-1 integrase detection during the course of cell infection, we took advantage of an infectious HIV-1 viral clone carrying IN tagged at the C-terminus with the HA epitope (HIV-1_IN-HA_[68]). Epitope fusion at the C-terminus of IN disrupted the *Vif* open reading frame. However, viral replication is *Vif*-independent in SupT1 human lymphocytic cells used in our study [69]. Using this virus, we first analyzed IN stability during the early steps of HIV-1 replication. Indeed, the ubiquitin degradation pathway targets IN to the proteasome [70], which may account for its short half-life in transiently transfected cells [70,71]. SupT1 cells were exposed to HIV-1 for 2 h and harvested at 2 h, 4 h and 6 h p.i. At each time whole cell extracts (WCE) were prepared and viral proteins CA, MA and IN-HA were detected by Western blotting. By optimizing cell infection conditions in order to maximize cell-virus contact surface, incoming CA, MA and IN-HA were readily detectable in cell extracts of infected cells. Infection of cells with a heat inactivated virus prevented detection of viral proteins in cells extracts, indicating that we specifically detected intracellular-associated viral proteins rather than virus absorbed at the cell surface (Figure 1A). Strikingly, the amount of IN-HA dramatically decreased during the 6 h period of time, whereas amounts of CA remained stable (Figure 1A and 1B). To ensure that the HA-tag did not interfere with IN properties, we compared the stability of HA-tagged and non-tagged IN proteins. SupT1 cells were infected either with wild type HIV-1 or HIV-1_IN-HA_, and IN was detected in cell lysates at different times post-infection using an anti-IN antibody. Regardless of the presence of a C-terminus HA-tag fusion, IN levels in cell lysates rapidly decreased to become barely detectable at 6 h p.i. (Figure 1B). In addition, levels of late reverse transcription product (LRT), 2-LTR circles and integrated viral DNA were similar at 6 h p.i. for both viruses indicating that the HA tag does not interfere with IN functions during early steps of virus replication (data not shown). Treatment of cells with proteasome inhibitor MG-132 resulted in rapid stabilization of IN-HA in infected cells indicating that once IN entered in the cell, a subset of the protein is actively degraded in a proteasome-dependent manner (Figure 1C). We further analyzed early steps of viral replication of HIV-1_IN-HA_. Quantitative PCR analysis indicated that the maximum amount of integrated provirus was reached at 10 h p.i (Figure 1D). Taken together, our results showed rapid degradation of IN protein upon cell infection, coinciding with detection of integrated proviral forms at 6 h p.i. Therefore, we decided to focus our study on early time points (i.e. 2 h p.i. and 6 h p.i.) to monitor the dynamic of IN-containing complexes.

### Dynamic distribution of HIV-1 integrase complexes during infection

**Figure 2:**
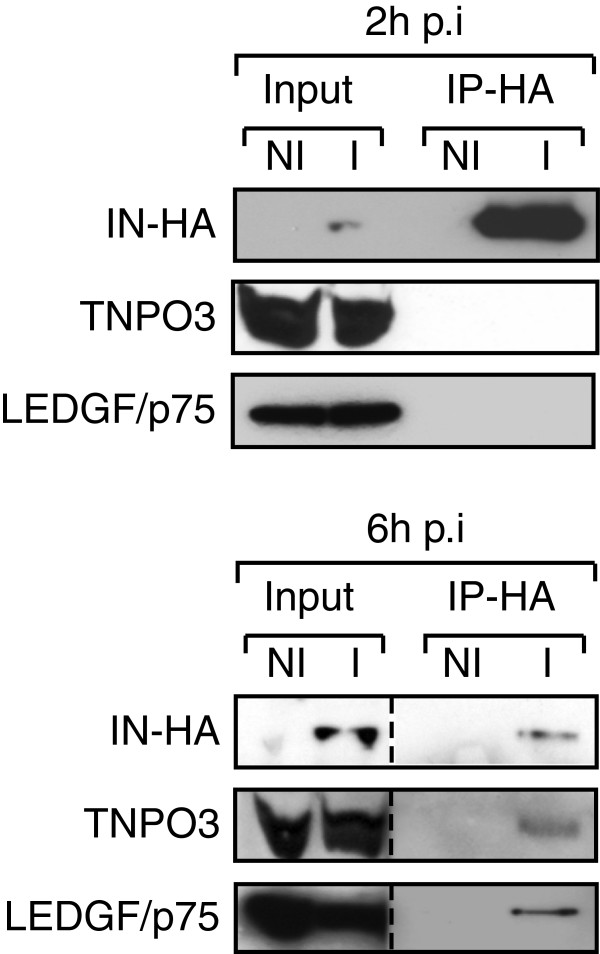
**IN interacts with cellular cofactors LEDGF/p75 and TNPO3 at 6 h p.i.** SupT1 cells were not infected (NI) or infected (I) with HIV-1_IN-HA_. WCE were prepared at 2 h and 6 h p.i. (as indicated), immunoprecipitated (IP-HA) with the anti-HA affinity matrix and analyzed by Western blotting using antibodies against HA, LEDGF/p75, and TNPO3. Inputs represent 5& of the immunoprecipitated material. To detect IN-HA at 2h p.i. the film was exposed for 5 minutes whereas detection of IN-HA at 6h p.i. required film exposure of 30 minutes. Dividing lines indicate the grouping of parts of the same gel.

**Figure 3:**
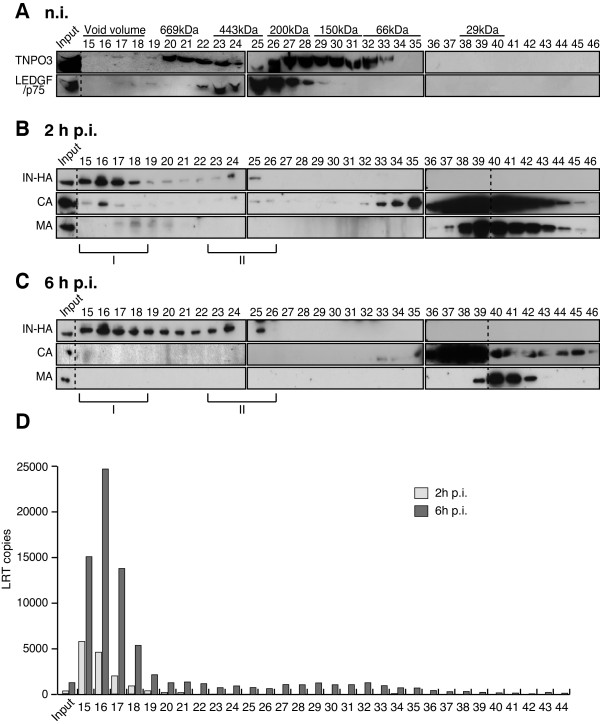
**Two distinct IN complexes are detected following cell infection. **SupT1 cells were either not infected (**A**) or infected with HIV-1_IN-HA_, and WCE were prepared at 2 h (**B**) and 6 h p.i. (**C**). WCE were subjected to gel filtration on a Superdex 200HR 10/300 column. Fractions were collected and analyzed by Western blotting using antibodies against LEDGF/p75, TNPO3, HA, MA, and CA. Inputs represent 3.5& of WCE load. Two complexes containing IN were detected, a high molecular weight complex (IN complex I, >1.3 MDa), and a low molecular weight complex (IN complex II, ~440KDa). To detect IN-HA at 2h p.i. in (**B**), the film was exposed for 5 minutes, whereas detection of IN-HA at 6h p.i. in (**C**) required film exposure of 30 minutes. (**D**) Viral DNA co-elutes with IN complex I. Same as (**A**) and (**B**) except DNA was extracted from each fraction and late reverse transcription product (LRT) content was quantified by real time PCR. Non-infected (NI) samples were used as controls (not shown). Results shown are representative of two independent experiments. Dividing lines indicate the grouping of parts of the different gels with identical times of film exposure.

The temporal dynamic of IN interactions with cellular cofactors during early steps of HIV replication was characterized by co-immunoprecipitation experiments. Interactions between IN-HA and host partners TNPO3 and LEDGF/p75 were readily detected at 6 h p.i. (Figure 2). To further assess the rapid changes of IN-associated complexes during the early stages of HIV-1 replication, we performed biochemical fractionation of infected cell lysates. First, fractions from size-exclusion chromatography of WCE were analyzed by Western blotting against TNPO3 and LEDGF/p75. In non-infected cells, the relatively broad elution profile of TNPO3 might reflect its interactions with several different proteins/cargos (fractions 17 to 33, Figure 3A). In contrast, a major peak around 300 KDa (Fractions 22–28, Figure 3A) was observed for LEDGF/p75. In addition, a small portion of LEDGF/p75 was found in higher molecular weight fractions (fractions 17–19, Figure 3A). Similar profiles were observed in infected cells (data not shown), indicating that HIV-1 infection does not trigger major changes in the distribution of LEDGF/p75 or TNPO3-associated complexes.

We then addressed the distribution of viral proteins early following infection. At 2 h p.i., IN-HA was detected in 2 complexes of different size and composition (Figure 3B). The high molecular weight complex (IN complex I), which was excluded from gel separation range (>1.3 MDa), was the most abundant one (fractions 15–18, Figure 3B). Viral proteins capsid (CA) and matrix (MA) were mostly found as free molecules (fractions 36–44, Figure 3B and C), except for a small proportion co-eluting with IN complex I (fractions 15–17 for CA and fraction 17–20 for MA, Figure 3B).

Although present in a minor proportion, low molecular weight (IN complex II) IN-HA complex peaked in fractions 23–25 (around 440 KDa). At 6 h p.i., IN-HA was still found in IN complex I, but shifted readily to IN complex II, while both CA and MA were only detected in smaller peaks (fraction 36–44) (Figure 3C). Noteworthy, IN-HA detection required longer exposure times at 6 h than at 2 h p.i., due to its rapid degradation upon cell infection. Together, our results indicate that shortly after infection, IN associates with at least two distinct complexes.

### IN low molecular weight complexes are devoid of viral DNA and *in vitro* PIC activity

**Figure 4:**
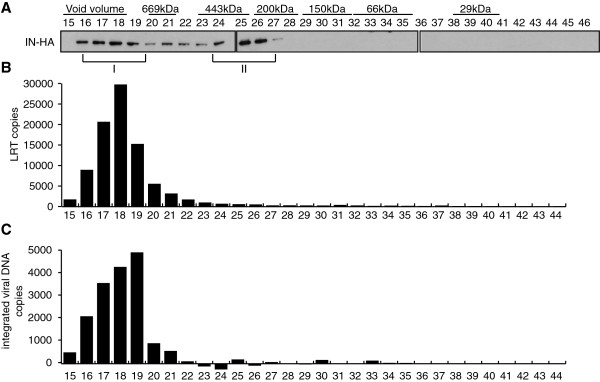
**Retroviral DNA and integration activity co-eluted with IN complex I.** SupT1 cells were infected with HIV-1_IN-HA _pseudotyped with VSV-G to increase infectivity. WCE were prepared at 6 h p.i. and subjected to gel filtration. (**A**) Fractions were collected and analyzed by Western blotting using antibody against HA. (**B**) DNA was extracted from each fraction and late reverse transcription product (LRT) content was quantified by real time PCR. (**C**) *In vitro* PIC activity was assayed for each fraction (see Methods for details).

To detect viral DNA potentially associated with IN complexes at these time points, we performed real-time PCR on DNA extracted from fractionated WCE (Figure 3D). At 2 h and 6 h p.i., viral DNA was detected in fractions 13 to 19 containing the IN complex I (Figure 3D). Elution of the viral cDNA in the void volume of the column (>1.3 MDa) is consistent with its estimated molecular weight (~6.4 MDa for the 9.7 kbp genome). *In vitro* integration activities of IN-containing complexes eluted from the column were also measured at 6 h p.i. To ensure optimal infection conditions, SupT1 cells were infected with VSV-G pseudotyped HIV-1_IN-HA_ and the ability of viral DNA to integrate into target plasmid *in vitro* was quantified by real-time PCR. Concomitant with the detection of viral DNA and IN in fractions containing complex I (Figure 4A and 4B), maximal PIC activity was also reached in these fractions (Figure 4C). Thereby, these data suggest that between 2 h and 6 h p.i. a significant portion of IN accumulates in a low molecular weight complex devoid of viral DNA.

### Accumulation of IN complex II does not require reverse transcription nor integration but depends on LEDGF/p75 expression

**Figure 5:**
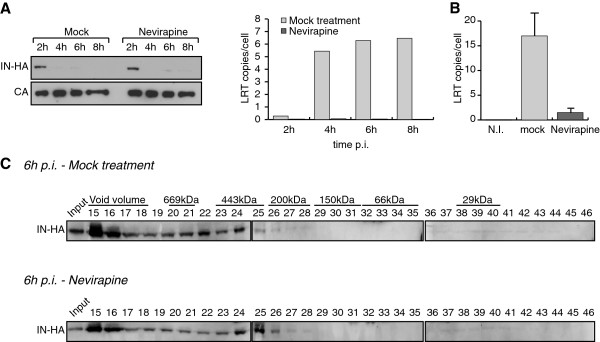
**Inhibition of reverse transcription does not affect the accumulation of IN complex II. (A) **IN is rapidly degraded in presence of Nevirapine. SupT1 cells were infected with HIV-1_IN-HA _in absence or presence of 1 μM of Nevirapine. At indicated time post infection, cells were lysed and analyzed using Western blotting with antibodies against HA and CA. The inhibition of reverse transcription was monitored in cell extracts by the quantification of late reverse transcription products (LRT) by real time PCR. **(B) **and **(C)** SupT1 cells were infected with HIV-1_IN-HA _in absence or presence of 1 μM of Nevirapine. The inhibition of reverse transcription was monitored in cell extracts by the quantification of late reverse transcription products (LRT) by real time PCR **(B)**. WCE were prepared at 2 h p.i. and subjected to gel filtration. Fractions were collected and analyzed by Western blotting using antibody against HA **(C)**.

To assess the role of reverse transcription in the accumulation of IN complex II, fractionation of infected cell extracts were conducted in the presence or absence of the non-nucleoside reverse transcriptase inhibitor Nevirapine. Nevirapine treatment did not affect the kinetics of IN degradation (Figure 5A). Furthermore, upon reverse transcription inhibition, IN was still detected in complex II (Figure 5B and 5C), suggesting that the accumulation of IN in low molecular weight complexes does not require the maturation of the reverse transcription complex (RTC) into a PIC.

**Figure 6:**
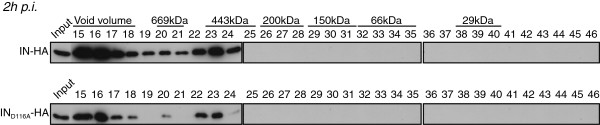
**IN complex II does not require integration. **SupT1 cells were infected with wild type HIV-1_IN-HA _or HIV-1_IN D116A-HA _harboring a mutation of integrase active site residue Asp116. WCE were prepared at 2 h p.i. and subjected to gel filtration. Fractions were collected and analyzed by Western blotting using antibody against HA.

Next, we tested whether IN complex II could be the result of a post-integration event leading to the release of DNA-free IN from integrated intasomes. SupT1 cells were infected with a virus harboring a catalytically inactivated IN_D116A_. At 2 h p.i., cell extracts were fractionated by size exclusion chromatography and collected fractions were analyzed by Western blotting. IN from HIV-1_IN D116A-HA_ eluted in two separate complexes (I and II) that were indistinguishable from the complexes obtained with WT IN (Figure 6), indicating that accumulation of IN complex II is not a post-integration event.

**Figure 7:**
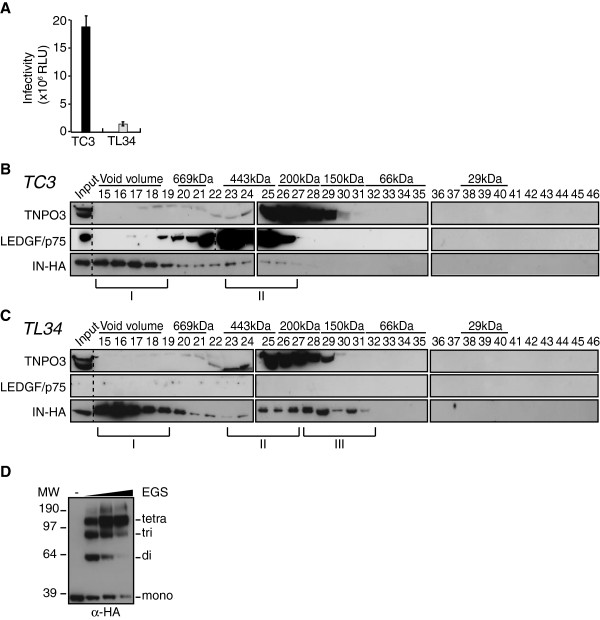
**IN complex II depends on LEDGF/p75. **(**A**) LEDGF/p75-knock-down SupT1 cells (TL34) and control SupT1 cells (TC3) were infected with VSV-G a pseudotyped HIV-1Δenv-Luc. 48 h p.i. cell lysates were analyzed for luciferase activity. Control polyclonal SupT1 cells (TC3) (**B**) or LEDGF/p75 knock-down TL34 cells (**C**) were infected with HIV-1_IN-HA_. WCE were prepared at 2 h p.i. and subjected to gel filtration on a Superdex 200HR 10/300 column. Fractions were collected and analyzed by Western blotting using antibodies against LEDGF/p75, TNPO3 and HA. Inputs represent 3,5& of WCE load. IN complexes I and II were detected in both TC3 and TL34 cells; yet a third complex with a lower molecular weight is detected only in TL34 cells (IN complex III, ~150KDa). Dividing lines indicate the grouping of parts of the different gels with identical times of film exposure. (**D**) Fractions 30-32, corresponding to part of the IN complex III, were pooled and proteins were cross-linked with increasing concentrations (0.25 to 2 mM) of the cross-linker ethylene glycol bis-succinimidylsuccinate (EGS). Cross-linked products were separated by SDS-PAGE and immunoblotted with anti-HA-HRP antibody.

We then investigated whether the distribution of the fractions containing IN was dependent on LEDGF/p75 expression. A LEDGF/p75-knock-down SupT1 cell clone (TL34) and its control polyclonal cell line counterpart (TC3) [72] were infected with VSV-G pseudotyped HIV-1Δenv-Luc. As expected, knock-down of endogenous LEDGF/p75 yielded a 10-fold decrease of viral infectivity, quantified by the viral encoded luciferase activity (Figure 7A). Then, WCE from TC3 or TL34 cells infected with HIV-1_IN-HA_ virus for 2 h were fractionated by size exclusion chromatography and collected fractions were analyzed by Western blotting. Consistently, we observed similar IN complexes (I and II) in TC3 lysates as in wild type SupT1 lysates (compare Figure 7B with 3B). In sharp contrast, in TL34 cells knocked down for LEDGF/p75 we observed a shift of IN complex II towards a lower molecular weight complex (IN complex III) with a molecular mass around 150 KDa (fractions 27–31) (Figure 7C). Regarding the ability of IN to form stable tetramers when expressed in human cells [51], we decided to further characterize IN complex III. We thus pooled fractions 30–32 and performed proteins cross-linking with increasing amount of the cross-linker ethylene glycol bis-succinimidylsuccinate (EGS). Addition of 0.25 to 2 mM of EGS yielded cross-linked complexes of 60, 90 and 130 KDa, with a strong predominance of the latter band at the higher concentrations of cross-linker agent (Figure 7D). Thus, depletion of LEDGF/p75 led to the accumulation of IN complex III that contains tetramers of IN.

### IN complexes II and III are exclusively nuclear

**Figure 8:**
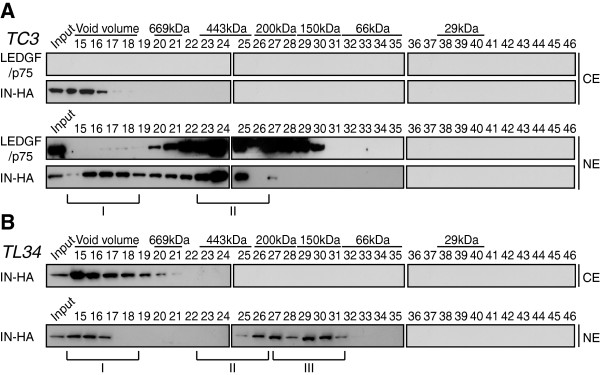
**IN complex II and III are exclusively nuclear. **Control SupT1 cells TC3 (**A**) and LEDGF/p75-knock-down SupT1 cells (TL34) (**B**) were infected with HIV-1_IN-HA_. Cytosolic and nuclear extracts (CE and NE, respectively) were prepared at 2 h p.i. and subjected to gel filtration on a Superdex 200HR 10/300 column. Fractions were collected and analyzed by Western blotting using antibodies against LEDGF/p75, or HA. Inputs represent 3,5& of WCE load. LEDGF/p75 served as an internal control for the nuclear fraction in (**A**).

We analyzed the cellular localization of the IN complexes at 2 h p.i. Cytosolic (CE) and nuclear extracts (NE) prepared from infected control (TC3) or LEDGF/p75-knock-down SupT1 cells (TL34) were fractionated. LEDGF/p75 was only detected in nuclear fractions of control cells, indicative of absence of NE contamination of the CE (Figure 8A). In both TC3 and TL34 cells, IN complex I was distributed between cytosolic and nuclear fractions, indicating that PICs reached the nuclear compartment as early as 2 h p.i. (Figure 8A and 8B). In sharp contrast, IN complex II was strictly associated with nuclear fractions and could not be detected in cytosolic extracts of TC3 cells (Figure 8A). Concordantly, in absence of LEDGF/p75, IN complex III was also only detected in nuclear fractions (Figure 8B). Taken together, our data indicate that IN complex II associates with nuclear fractions in a LEDGF/p75-dependent manner.

### A class II IN mutant virus failed to accumulate in low molecular weight complexes

**Figure 9:**
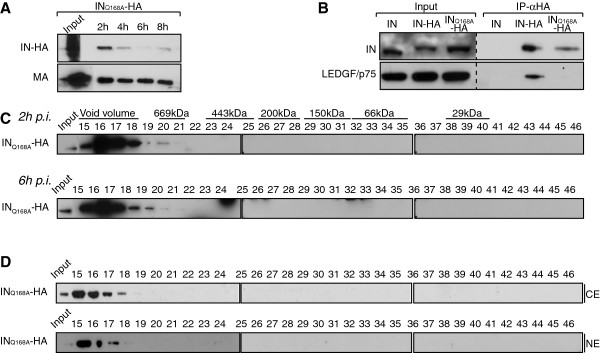
**IN Q168A failed to accumulate in a nuclear low molecular weight complex. **(**A**) IN _Q168A_-HA levels rapidly decreased following viral infection. SupT1 cells were infected with HIV-1_IN Q168A-HA _for 2 h. At indicated time post infection, cells were lysed and equivalent amounts of each sample (100 μg of protein) were analyzed using Western blotting with antibodies against HA and MA. Input represents 0.1& of the amount of virus used to infect the cells. **(B) **SupT1 cells were infected with HIV-1 viruses encoding WT IN, IN-HA or IN_Q168A_-HA. IN-HA was immunoprecipitated with the anti-HA affinity matrix and analyzed by Western blotting using antibodies against HA and LEDGF/p75. Inputs represent 5& of the immunoprecipitated material. **(C) **SupT1 cells were infected with HIV-1_IN D116A-HA_ and WCE prepared at 2 h and 6 h p.i. were subjected to gel filtration. Fractions were collected and analyzed by Western blotting using antibodies against HA. Inputs represent 3.5& of WCE load. **(D) **Cytosolic and nuclear extracts (CE and NE, respectively) were prepared at 2 h p.i., subjected to gel filtration and collected fractions were analyzed by Western blotting using antibody against HA. Inputs represent 3,5& of WCE load.

To further explore the mechanisms of the accumulation of IN in low molecular weight complexes, we took advantage of an IN class II mutant virus HIV-1_IN__Q168A-HA_ that was first identified to be defective for LEDGF/p75 interaction [52]. Mutation of the Q168 residue of IN impairs its ability to form tetramers, resulting in a decrease of its concerted integration activity [43,73]. First, the stability of the mutant IN _Q168A_ was assessed during infection. WCE from SupT1 cells infected with HIV-1_IN__Q168A-HA_ were harvested at 2 h, 4 h, 6 h and 8 h p.i. and viral proteins MA and IN were detected by Western blotting. IN_Q168A_ levels decreased rapidly during the early phase of HIV-1 infection suggesting that, similar to the WT IN, this mutant was degraded by the proteasome (Figure 9A). Next, the interaction between IN and LEDGF/p75 was assessed in cells infected with HIV-1 encoding wild type IN devoid of the HA tag, IN-HA or IN_Q168A_-HA. As expected, the Q168A mutation ablated the LEDGF/p75-IN interaction (Figure 9B). Surprisingly, fractionation of infected WCE indicated that IN_Q168A_ failed to accumulate in low molecular weight complexes at 2 h and 6 h p.i. (Figure 9C). However, the high molecular weight complexes eluting in the void volume of the column (IN complex I) is still associated both with the cytoplasm and the nuclear fractions of the cells (Figure 9D). Taken together, these results indicated that the nuclear accumulation of IN complex II is impaired for a mutant defective for LEDGF/p75 interaction and tetramerization.

## Discussion

Studies of retroviral pre-integration complexes have been hampered by the limited amounts available from infected cells. In this study, we took advantage of an infectious HIV-1 viral clone carrying a C-terminus HA-tagged IN protein to explore IN low molecular weight complexes shortly after cell entry.

Our results show that IN undergoes rapid degradation by the proteasome shortly after its entry in the cytoplasm, whereas CA and MA remained stable. Previous published data showed that HIV-1 IN over-expressed in cells is actively degraded by the ubiquitin/proteasome system in both N-end rule dependent and independent pathways [52,70,71,74-76]. Furthermore, the binding to LEDGF/p75 was shown to stabilize IN by preventing its degradation by the proteasome [52,75]. However, we observed a similar decrease of levels of the WT IN and the Q168A mutant impaired for LEDGF/p75 interaction. This is likely to reflect that at early steps of infection the vast majority of IN entering the cell cytoplasm is not yet accessible for LEDGF/p75 binding.

In preliminary studies aiming at analyzing the impact of HIV-1 infection on cellular cofactors complexes using a Superdex 200 10/300 GL column, we noticed that IN eluted in a low molecular weight complex that was distinct from the large IN-containing complex detected within the void volume of the gel filtration column. This large complex also co-eluted with viral DNA and integration activity, indicative of the presence of active PICs in these fractions. This observation is consistent with previous characterizations of retroviral nucleoprotein complexes in infected cells using gel filtration chromatography [2,6,21,25]. In contrast, the low molecular weight complex containing IN was devoid of viral cDNA and integrase activity. This complex was not observed in previous studies of HIV-1 PIC composition using gel filtration chromatography [21] or fractionation by sucrose gradient [77]. However, this is not surprising as these analyses were restricted to the cytoplasmic fraction of infected cells, precluding the detection of complex II that we found to be exclusively in the nuclear fraction. IN complex II could be similar to the one that was previously isolated from the nucleus of cells stably expressing IN. In this report, tetramers of INs associated with LEDGF/p75 were found in a complex with an estimated mass around 400 KDa [51]. Accordingly, we found that depletion of LEDGF/p75 lead to a shift towards a lower molecular weight complex (IN complex III) that peaks at ~150 KDa and contains tetramers of IN.

Non-exclusive hypothesis could explain the presence of IN in a low molecular weight complex distinct from the PIC. First, IN complex II could be the result of PIC destabilization. Indeed, the relative increase of the proportion of IN complex II at 6 h p.i. could reflect a dynamic shift of IN from active PICs to lower molecular weight complexes lacking full-length viral cDNA. Several mechanisms could account for the escape of IN from active PICs. Inefficient reverse transcription [78,79], or degradation of incoming viral cDNA by cytosolic exonucleases like TREX1 [80] could lead to the destabilization of PICs and the release of free IN. However, we did not observe a significant change in the distribution of IN complexes I and II upon inhibition of reverse transcription by the non-nucleoside reverse transcriptase inhibitor Nevirapine, challenging the hypothesis that IN complex II formation is the result of abortive reverse transcription complexes. Moreover, a catalytic inactive mutant IN_D116A_ accumulated in complex II suggesting that it is not the result of a post-integration event. Further experiments will be required to assess the potential role of cellular exonucleases in the release of low molecular weight IN complexes distinct from the PICs.

A second hypothesis posits that IN complex II might originate from IN molecules that are not associated with the PIC. IN is incorporated in virions as part of the Gag-Pol polyprotein precursor, and it is generally admitted that between 30 to 100 of IN molecules are packaged per retroviral particle [81]. Although a precise quantification of IN incorporated into HIV-1 particles is still being awaited, it is likely that IN is present in large excess. Indeed, the functional intasome is formed by tetramer of IN tightly bound to the LTR ends of the newly synthesized viral cDNA [82,83]. Thus, this apparent excess of DNA-free IN could lead to the formation of IN complex II. Future experiments will reveal whether IN low molecular weight complexes play an active role during HIV-1 replication.

## Conclusions

We show that, shortly after the virus enters the cell, a significant portion of IN distinct from active PICs forms a low molecular weight complex in the nucleus that is dependent on LEDGF/p75.

## Methods

### Cells and viruses

293 T cells were grown in DMEM plus glutamine, antibiotics and 10& decomplemented-FCS (foetal calf serum) (GibcoBRL, Invitrogen). SupT1, TC3 and TL34 cells were grown in RPMI 1640 plus glutamine, antibiotics and 10& decomplemented-FCS (foetal calf serum) (GibcoBRL, Invitrogen). Virus stocks were generated by transfecting 293 T cells with either wild type or IN-HA tagged Lai molecular clone (HIV-1_IN-HA_[68]) using Fugene 6 reagent (Roche). HIV-1_IN__Q168A-HA_ and HIV-1_IN D116A-HA_ were generated by site-directed mutagenesis using HIV-1_IN-HA_ as a template. Mutations were confirmed by sequencing and subcloned back into the PflMI sites of the WT HIV-1_IN-HA_. HIV-1 Δ*env* Luc has been previously described [84]. VSV-G pseudotyped viruses were produced by co-transfecting HIV-1_IN-HA_ or HIV-1 Δ*env* Luc with plasmid pMD.G [85]. Twenty-four hours post-transfection, cells were washed with PBS and supernatants were collected at 48 h and 72 h post-transfection. The supernatants were 0.45-μm-filtered and ultracentrifuged at 150,000 *g* for 1 h 30 minutes at 4°C. Virus pellets from 30 ml of supernatants were resuspended in 75 μl of PBS. An equal volume of RPMI 1640 medium (GIBCO, Invitrogen) supplemented with 10& fetal calf serum and antibiotics was added, and virus stocks were stored at −80°C. HIV-1 CAp24 antigen was quantified by ELISA (Innotests HIV Antigen mAb, Innogenetics, France).

### Cell infection and cell extract preparation

SupT1 cells (2 × 10^8^) were infected with 200 μg of CAp24 (corresponding to a multiplicity of infection-MOI- of 5, as quantified by real time PCR) of HIV-1 or HIV-1 _IN-HA_ virus in a total volume of 500 μl for 2 h at 37°C. When indicated, 20 μM of MG-132 was added along the course of infection. Two hours later, cells were washed three times in 25 ml of PBS and resuspended in RPMI 1640 medium supplemented with 10& fetal calf serum and antibiotics at a final concentration of 1.5 × 10^6^ cells/ml. At different time post infection cells were harvested, washed twice with 25 ml of PBS and lysed in 3 cell pellet volumes of lysis buffer (20 mM Tris–HCl pH 8.0, 0.3 M KCl, 5 mM MgCl_2_, 10& (v/v) glycerol, 0.1& tween 20, 1 mM PMSF and protease inhibitor cocktail from Sigma). Cell lysis was completed by two successive rounds of freeze-thaw, then incubated for 30 min at 4°C on rotating wheel. Two successive centrifugation steps at 16,000 *g* for 30 min at 4°C allowed complete removal of insoluble materials. The collected supernatant corresponding to soluble proteins within the cells was called whole cell extracts (WCE). Cytosolic and nuclear extracts were obtained by sequential cell fractionation. Cells were first washed in buffer A (20 mM HEPES-KOH pH 7.9, 1.5 mM MgCl_2_, 10 mM KCl, 1 mM PMSF and protease inhibitor cocktail from Sigma) and lysed 5 min on ice in 2 cell pellet volumes in buffer A and 0.1& (v/v) NP-40. After centrifugation of the cell lysate at 10,000 *g* for 5 min, the supernatant corresponding to the cytosolic extract (CE) was collected and the pellet was resuspended in 3 volumes of buffer B (20 mM HEPES-KOH pH 7.9, 1.5 mM MgCl_2_, 0.5 M NaCl, 25& (v/v) glycerol, 1 mM PMSF and protease inhibitor cocktail from Sigma). After 15 minutes incubation on ice, the nuclear extract (NE) was collected by centrifugation for 15 minutes at 14,000 *g*. CE and NE were stored at −80°C.

### PIC activity assay

The assay is based on the quantification of integration events of viral cDNA into a parental vector (pTZ-19R) [8] with the following modifications. SupT1 cells were infected for 2 h with a VSV-G pseudotyped HIV-1_IN-HA_ virus and WCE were prepared at 6 h p.i. as previously described. WCE (3 mg) were injected into Superdex 200 10/300 GL gel filtration column. Each fraction was collected and treated with RNase A (final concentration at 20 μg/ml) for 20 minutes at RT in a final volume of 250 μl. Integration reaction assay was performed at 37°C for 45 minutes by mixing each fraction with 1 μg of target plasmid pTZ-19R in integrase reaction buffer (INRB: 20 mM HEPES-KOH pH 7.4, 150 mM KCl, 1 mM MgCl_2_, 4& glycerol, and 1 mM DTT added just before starting the reaction) in a final volume of 350 μl. For each fraction, negative control was performed by omitting the target plasmid in the integration reaction assay. The reaction was stopped by adding 0.5& SDS, 8 mM EDTA and 0.5 mg/ml proteinase K for 1 h at 56°C. DNA was then extracted with phenol and phenol:chloroform:isoamylalcohol 25:24:1, using glycogen as carrier, and stored at −20°C. Integration events were quantified using a two-step PCR reaction. In the first round PCR, integrated HIV-1 sequences were amplified using the HIV-1 specific LM667 primer [78] together with primers annealing to opposite strand of pTZ-19R in tail-to-tail fashion. TZ2414 primer sequence is 5′- GTTGTTCCAGTTTG GAACAAGAGTC-3′. TZ2413 primer sequence is 5′- ACTCAACCCTATCTCGGTCTATTC-3′. To evidence background PCR signal, TZ2414 and TZ2413 primers were omitted from each integration reaction. We performed the second nested-PCR steps using conditions previously described [78]. Results shown are indicated as relative units of specific signal (signal from PCR with target and with TZ2414/TZ2413 primers) minus unspecific signal (signal from PCR without target or without TZ2414/TZ2413 primers). The copy number of integrated DNA was determined in reference to a standard curve obtained by concomitant two steps amplification of serial dilution of the standard pNLX-HIVLTR vector. This construct was generated by amplifying the LTR region (n.t. 1–702, numbering based on NL4.3 sequence) and cloning into BamH1 and Pst1 restriction endonuclease sites of pTZ-19R.

### Quantification of viral cDNA by real-time PCR

Prior to infection, viral stocks were treated 1 h at 37°C with 100 U per ml of DNAseI (Roche applied Science). SupT1 cells (6x10^6^) were infected with viral doses corresponding to 6 μg of HIV-1_IN-HA_ CAp24 antigen in 12-wells plates. At 2 h p.i., cells were washed twice in PBS. At 2 h, 4 h, 6 h, 8 h and 10 h p.i. cells were harvested, washed twice in PBS and DNA was extracted using the QIAamp Blood DNA Minikit (Qiagen). Quantifications of viral DNA were performed by real-time PCR using the LightCycler 480 system (Roche Applied Science). Primers, probes and PCR run conditions were described previously [84]. The copy numbers of HIV-1 late reverse transcription product (LRT) and 2-LTR circles were determined using standard curves obtained by amplification of cloned DNA containing the matched sequences. The copy number of integrated HIV-1 DNA was determined in reference to a standard curve generated by concomitant two-stage PCR amplification of a serial dilution of the standard HeLa HIVR7-Neo cell DNA [78]. Copy numbers of each viral form were normalized with the number of cells obtained by the quantification by PCR of the β-globin gene according to the manufacturer instructions (Roche Applied Science).

### Immunoprecipitation

For co-immunoprecipitation experiments, 3 mg of WCE were mixed with 50 μl (50& slurry) of anti-HA affinity matrix (clone 3 F10, Roche Applied Science) supplemented with protease inhibitor cocktail and 1 mM PMSF for 3 h at 4°C on rotating wheel. Beads were washed three times with 15 volumes PBS-0.1& tween 20 for 5 minutes on a rotating wheel at 4°C. IN-HA complexes were directly resuspended in 1× loading sample buffer and boiled for 5 min.

### Chromatography

Whole cell extracts were subjected to size exclusion chromatography at 4°C using an AKTA purifier system (GE Healthcare). Four mg of WCE were injected on a Superdex 200 10/300 GL column and 400 μl fractions were collected at a flow rate of 0.5 ml/min of WCE buffer. Proteins were precipitated over-night at 4°C in 10& trichloroacetic acid, washed twice in cold acetone and analyzed by Western blotting. Protein standards (Sigma) were fractionated under same conditions to estimate the size of the eluted protein complexes. When required, DNA was extracted from each fraction using the QIAamp Blood DNA Minikit (Qiagen).

### Cross-linking

Cross-linking reactions were performed on gel filtration fractions collected in 20 mM HEPES-KOH pH 7.2, 0.3 M KCl, 5 mM MgCl_2_, 10& glycerol, 0.1& tween 20, 1 mM PMSF and protease inhibitor cocktail (Sigma). Fractions 30–32, corresponding to part of IN complex III, were pooled and proteins were cross-linked with 0.25 mM to 2 mM of the cross-linker ethylene glycol bis-succinimidylsuccinate (EGS, freshly prepared) for 30 minutes at room temperature. The reaction was stopped by adding Tris–HCl pH 7.5 at a final concentration of 50 mM for 15 minutes at room temperature.

### SDS-Page and Western-blotting

Proteins were separated by 4-12& gradient SDS-PAGE (Invitrogen), transferred onto nitrocellulose membranes, and revealed by Western-blotting using the following antibodies as indicated: anti-CAp24 (AIDS Research and Reference Reagent Program), anti-IN (Santa Cruz), anti-MA (Hybridolab), anti-HA-HRP (clone 3 F10, Roche Applied Science), LEDGF/p75 (BD Bioscience), TNPO3 (Abcam), and α-tubulin (Sigma-Aldrich).

## Competing interests

The authors declare that they have no competing interests.

## Authors’ contributions

AG, MB and SE designed experiments; AG, NS, ES and SE performed experiments; AG, NS and SE analyzed the data; AG and SE wrote the paper, and AG, NS and SE commented and corrected on manuscript drafts. All authors read and approved the final manuscript.

## References

[B1] Nisole S Saib A Early steps of retrovirus replicative cycle Retrovirology 2004 1 9 10.1186/1742-4690-1-9 15169567 PMC421752

[B2] Bowerman B Brown PO Bishop JM Varmus HE A nucleoprotein complex mediates the integration of retroviral DNA Genes Dev 1989 3 469 478 10.1101/gad.3.4.469 2721960

[B3] Brown PO Bowerman B Varmus HE Bishop JM Correct integration of retroviral DNA in vitro Cell 1987 49 347 356 10.1016/0092-8674(87)90287-X 3032450

[B4] Fujiwara T Mizuuchi K Retroviral DNA integration: structure of an integration intermediate Cell 1988 54 497 504 10.1016/0092-8674(88)90071-2 3401925

[B5] Ellison V Abrams H Roe T Lifson J Brown P Human immunodeficiency virus integration in a cell-free system J Virol 1990 64 2711 2715 2335814 10.1128/jvi.64.6.2711-2715.1990 PMC249450

[B6] Farnet CM Haseltine WA Integration of human immunodeficiency virus type 1 DNA in vitro Proc Natl Acad Sci U S A 1990 87 4164 4168 10.1073/pnas.87.11.4164 2349226 PMC54068

[B7] Hansen MS Bushman FD Human immunodeficiency virus type 2 preintegration complexes: activities in vitro and response to inhibitors J Virol 1997 71 3351 3356 9060709 10.1128/jvi.71.4.3351-3356.1997 PMC191478

[B8] Engelman A Isolation and analysis of HIV-1 preintegration complexes Methods Mol Biol 2009 485 135 149 19020823 10.1007/978-1-59745-170-3_10

[B9] Bushman FD Fujiwara T Craigie R Retroviral DNA integration directed by HIV integration protein in vitro Science 1990 249 1555 1558 10.1126/science.2171144 2171144

[B10] Craigie R Fujiwara T Bushman F The IN protein of Moloney murine leukemia virus processes the viral DNA ends and accomplishes their integration in vitro Cell 1990 62 829 837 10.1016/0092-8674(90)90126-Y 2167180

[B11] Engelman A Mizuuchi K Craigie R HIV-1 DNA integration: mechanism of viral DNA cleavage and DNA strand transfer Cell 1991 67 1211 1221 10.1016/0092-8674(91)90297-C 1760846

[B12] Chen H Wei SQ Engelman A Multiple integrase functions are required to form the native structure of the human immunodeficiency virus type I intasome J Biol Chem 1999 274 17358 17364 10.1074/jbc.274.24.17358 10358097

[B13] Wei SQ Mizuuchi K Craigie R A large nucleoprotein assembly at the ends of the viral DNA mediates retroviral DNA integration EMBO J 1997 16 7511 7520 10.1093/emboj/16.24.7511 9405379 PMC1170350

[B14] Wei SQ Mizuuchi K Craigie R Footprints on the viral DNA ends in moloney murine leukemia virus preintegration complexes reflect a specific association with integrase Proc Natl Acad Sci U S A 1998 95 10535 10540 10.1073/pnas.95.18.10535 9724738 PMC27929

[B15] Brown PO Bowerman B Varmus HE Bishop JM Retroviral integration: structure of the initial covalent product and its precursor, and a role for the viral IN protein Proc Natl Acad Sci U S A 1989 86 2525 2529 10.1073/pnas.86.8.2525 2539592 PMC286949

[B16] Pauza CD Two bases are deleted from the termini of HIV-1 linear DNA during integrative recombination Virology 1990 179 886 889 10.1016/0042-6822(90)90161-J 2238479

[B17] Yoder KE Bushman FD Repair of gaps in retroviral DNA integration intermediates J Virol 2000 74 11191 11200 10.1128/JVI.74.23.11191-11200.2000 11070016 PMC113210

[B18] Vincent KA York-Higgins D Quiroga M Brown PO Host sequences flanking the HIV provirus Nucleic Acids Res 1990 18 6045 6047 10.1093/nar/18.20.6045 2235486 PMC332403

[B19] Bukrinsky MI Sharova N McDonald TL Pushkarskaya T Tarpley WG Stevenson M Association of integrase, matrix, and reverse transcriptase antigens of human immunodeficiency virus type 1 with viral nucleic acids following acute infection Proc Natl Acad Sci U S A 1993 90 6125 6129 10.1073/pnas.90.13.6125 7687060 PMC46880

[B20] Farnet CM Bushman FD HIV-1 cDNA integration: requirement of HMG I(Y) protein for function of preintegration complexes in vitro Cell 1997 88 483 492 10.1016/S0092-8674(00)81888-7 9038339

[B21] Farnet CM Haseltine WA Determination of viral proteins present in the human immunodeficiency virus type 1 preintegration complex J Virol 1991 65 1910 1915 2002549 10.1128/jvi.65.4.1910-1915.1991 PMC240011

[B22] Gallay P Swingler S Song J Bushman F Trono D HIV nuclear import is governed by the phosphotyrosine-mediated binding of matrix to the core domain of integrase Cell 1995 83 569 576 10.1016/0092-8674(95)90097-7 7585960

[B23] Iordanskiy S Berro R Altieri M Kashanchi F Bukrinsky M Intracytoplasmic maturation of the human immunodeficiency virus type 1 reverse transcription complexes determines their capacity to integrate into chromatin Retrovirology 2006 3 4 10.1186/1742-4690-3-4 16409631 PMC1360674

[B24] Karageorgos L Li P Burrell C Characterization of HIV replication complexes early after cell-to-cell infection AIDS Res Hum Retroviruses 1993 9 817 823 10.1089/aid.1993.9.817 7504934

[B25] Miller MD Farnet CM Bushman FD Human immunodeficiency virus type 1 preintegration complexes: studies of organization and composition J Virol 1997 71 5382 5390 9188609 10.1128/jvi.71.7.5382-5390.1997 PMC191777

[B26] Li L Yoder K Hansen MS Olvera J Miller MD Bushman FD Retroviral cDNA integration: stimulation by HMG I family proteins J Virol 2000 74 10965 10974 10.1128/JVI.74.23.10965-10974.2000 11069991 PMC113176

[B27] Li L Olvera JM Yoder KE Mitchell RS Butler SL Lieber M Martin SL Bushman FD Role of the non-homologous DNA end joining pathway in the early steps of retroviral infection EMBO J 2001 20 3272 3281 10.1093/emboj/20.12.3272 11406603 PMC150207

[B28] Lin CW Engelman A The barrier-to-autointegration factor is a component of functional human immunodeficiency virus type 1 preintegration complexes J Virol 2003 77 5030 5036 10.1128/JVI.77.8.5030-5036.2003 12663813 PMC152146

[B29] Llano M Vanegas M Fregoso O Saenz D Chung S Peretz M Poeschla EM LEDGF/p75 determines cellular trafficking of diverse lentiviral but not murine oncoretroviral integrase proteins and is a component of functional lentiviral preintegration complexes J Virol 2004 78 9524 9537 10.1128/JVI.78.17.9524-9537.2004 15308744 PMC506940

[B30] Suzuki Y Craigie R The road to chromatin - nuclear entry of retroviruses Nat Rev Microbiol 2007 5 187 196 10.1038/nrmicro1579 17304248

[B31] Arhel NJ Souquere-Besse S Munier S Souque P Guadagnini S Rutherford S Prevost MC Allen TD Charneau P HIV-1 DNA Flap formation promotes uncoating of the pre-integration complex at the nuclear pore EMBO J 2007 26 3025 3037 10.1038/sj.emboj.7601740 17557080 PMC1894778

[B32] Zennou V Petit C Guetard D Nerhbass U Montagnier L Charneau P HIV-1 genome nuclear import is mediated by a central DNA flap Cell 2000 101 173 185 10.1016/S0092-8674(00)80828-4 10786833

[B33] Yamashita M Emerman M The cell cycle independence of HIV infections is not determined by known karyophilic viral elements PLoS Pathog 2005 1 e18 10.1371/journal.ppat.0010018 16292356 PMC1283251

[B34] Yamashita M Perez O Hope TJ Emerman M Evidence for direct involvement of the capsid protein in HIV infection of nondividing cells PLoS Pathog 2007 3 1502 1510 17967060 10.1371/journal.ppat.0030156 PMC2042020

[B35] Brass AL Dykxhoorn DM Benita Y Yan N Engelman A Xavier RJ Lieberman J Elledge SJ Identification of host proteins required for HIV infection through a functional genomic screen Science 2008 319 921 926 10.1126/science.1152725 18187620

[B36] Christ F Thys W De Rijck J Gijsbers R Albanese A Arosio D Emiliani S Rain JC Benarous R Cereseto A Debyser Z Transportin-SR2 imports HIV into the nucleus Curr Biol 2008 18 1192 1202 10.1016/j.cub.2008.07.079 18722123

[B37] Konig R Zhou Y Elleder D Diamond TL Bonamy GM Irelan JT Chiang CY Tu BP De Jesus PD Lilley CE Global analysis of host-pathogen interactions that regulate early-stage HIV-1 replication Cell 2008 135 49 60 10.1016/j.cell.2008.07.032 18854154 PMC2628946

[B38] De Iaco A Luban J Inhibition of HIV-1 infection by TNPO3 depletion is determined by capsid and detectable after viral cDNA enters the nucleus Retrovirology 2011 8 98 10.1186/1742-4690-8-98 22145813 PMC3267670

[B39] Schaller T Ocwieja KE Rasaiyaah J Price AJ Brady TL Roth SL Hue S Fletcher AJ Lee K Kewalramani VN HIV-1 capsid-cyclophilin interactions determine nuclear import pathway. Integration targeting and replication efficiency PLoS Pathog 2011 7 e1002439 10.1371/journal.ppat.1002439 22174692 PMC3234246

[B40] Zhou L Sokolskaja E Jolly C James W Cowley SA Fassati A Transportin 3 promotes a nuclear maturation step required for efficient HIV-1 integration PLoS Pathog 2011 7 e1002194 10.1371/journal.ppat.1002194 21901095 PMC3161976

[B41] Kataoka N Bachorik JL Dreyfuss G Transportin-SR, a nuclear import receptor for SR proteins J Cell Biol 1999 145 1145 1152 10.1083/jcb.145.6.1145 10366588 PMC2133142

[B42] Rain JC Cribier A Gerard A Emiliani S Benarous R Yeast two-hybrid detection of integrase-host factor interactions Methods 2009 47 291 297 10.1016/j.ymeth.2009.02.002 19232540

[B43] Cribier A Segeral E Delelis O Parissi V Simon A Ruff M Benarous R Emiliani S Mutations affecting interaction of integrase with TNPO3 do not prevent HIV-1 cDNA nuclear import Retrovirology 2011 8 104 10.1186/1742-4690-8-104 22176773 PMC3286403

[B44] Krishnan L Matreyek KA Oztop I Lee K Tipper CH Li X Dar MJ Kewalramani VN Engelman A The requirement for cellular transportin 3 (TNPO3 or TRN-SR2) during infection maps to human immunodeficiency virus type 1 capsid and not integrase J Virol 2010 84 397 406 10.1128/JVI.01899-09 19846519 PMC2798409

[B45] Thys W De Houwer S Demeulemeester J Taltynov O Vancraenenbroeck R Gerard M De Rijck J Gijsbers R Christ F Debyser Z Interplay between HIV entry and transportin-SR2 dependency Retrovirology 2011 8 7 10.1186/1742-4690-8-7 21276267 PMC3041740

[B46] Cherepanov P Maertens GN Hare S Structural insights into the retroviral DNA integration apparatus Curr Opin Struct Biol 2011 21 249 256 10.1016/j.sbi.2010.12.005 21277766

[B47] Li X Krishnan L Cherepanov P Engelman A Structural biology of retroviral DNA integration Virology 2011 411 194 205 10.1016/j.virol.2010.12.008 21216426 PMC3640404

[B48] Ciuffi A Llano M Poeschla E Hoffmann C Leipzig J Shinn P Ecker JR Bushman F A role for LEDGF/p75 in targeting HIV DNA integration Nat Med 2005 11 1287 1289 10.1038/nm1329 16311605

[B49] Marshall HM Ronen K Berry C Llano M Sutherland H Saenz D Bickmore W Poeschla E Bushman FD Role of PSIP1/LEDGF/p75 in lentiviral infectivity and integration targeting PLoS One 2007 2 e1340 10.1371/journal.pone.0001340 18092005 PMC2129110

[B50] Shun MC Raghavendra NK Vandegraaff N Daigle JE Hughes S Kellam P Cherepanov P Engelman A LEDGF/p75 functions downstream from preintegration complex formation to effect gene-specific HIV-1 integration Genes Dev 2007 21 1767 1778 10.1101/gad.1565107 17639082 PMC1920171

[B51] Cherepanov P Maertens G Proost P Devreese B Van Beeumen J Engelborghs Y De Clercq E Debyser Z HIV-1 integrase forms stable tetramers and associates with LEDGF/p75 protein in human cells J Biol Chem 2003 278 372 381 12407101 10.1074/jbc.M209278200

[B52] Emiliani S Mousnier A Busschots K Maroun M Van Maele B Tempe D Vandekerckhove L Moisant F Ben-Slama L Witvrouw M Integrase mutants defective for interaction with LEDGF/p75 are impaired in chromosome tethering and HIV-1 replication J Biol Chem 2005 280 25517 25523 10.1074/jbc.M501378200 15855167

[B53] Turlure F Devroe E Silver PA Engelman A Human cell proteins and human immunodeficiency virus DNA integration Front Biosci 2004 9 3187 3208 10.2741/1472 15353349

[B54] Ge H Roeder RG Purification, cloning, and characterization of a human coactivator, PC4, that mediates transcriptional activation of class II genes Cell 1994 78 513 523 10.1016/0092-8674(94)90428-6 8062391

[B55] Ge H Si Y Roeder RG Isolation of cDNAs encoding novel transcription coactivators p52 and p75 reveals an alternate regulatory mechanism of transcriptional activation EMBO J 1998 17 6723 6729 10.1093/emboj/17.22.6723 9822615 PMC1171017

[B56] Yokoyama A Cleary ML Menin critically links MLL proteins with LEDGF on cancer-associated target genes Cancer Cell 2008 14 36 46 10.1016/j.ccr.2008.05.003 18598942 PMC2692591

[B57] Engelman A Cherepanov P The lentiviral integrase binding protein LEDGF/p75 and HIV-1 replication PLoS Pathog 2008 4 e1000046 10.1371/journal.ppat.1000046 18369482 PMC2275779

[B58] Poeschla EM Integrase, LEDGF/p75 and HIV replication Cell Mol Life Sci 2008 65 1403 1424 10.1007/s00018-008-7540-5 18264802 PMC3902792

[B59] Busschots K Voet A De Maeyer M Rain JC Emiliani S Benarous R Desender L Debyser Z Christ F Identification of the LEDGF/p75 binding site in HIV-1 integrase J Mol Biol 2007 365 1480 1492 10.1016/j.jmb.2006.10.094 17137594

[B60] Cherepanov P Sun ZY Rahman S Maertens G Wagner G Engelman A Solution structure of the HIV-1 integrase-binding domain in LEDGF/p75 Nat Struct Mol Biol 2005 12 526 532 10.1038/nsmb937 15895093

[B61] Rahman S Lu R Vandegraaff N Cherepanov P Engelman A Structure-based mutagenesis of the integrase-LEDGF/p75 interface uncouples a strict correlation between in vitro protein binding and HIV-1 fitness Virology 2007 357 79 90 10.1016/j.virol.2006.08.011 16959283

[B62] De Rijck J Vandekerckhove L Gijsbers R Hombrouck A Hendrix J Vercammen J Engelborghs Y Christ F Debyser Z Overexpression of the lens epithelium-derived growth factor/p75 integrase binding domain inhibits human immunodeficiency virus replication J Virol 2006 80 11498 11509 10.1128/JVI.00801-06 16987986 PMC1642583

[B63] Llano M Vanegas M Hutchins N Thompson D Delgado S Poeschla EM Identification and characterization of the chromatin-binding domains of the HIV-1 integrase interactor LEDGF/p75 J Mol Biol 2006 360 760 773 10.1016/j.jmb.2006.04.073 16793062

[B64] Vandekerckhove L Christ F Van Maele B De Rijck J Gijsbers R Van den Haute C Witvrouw M Debyser Z Transient and stable knockdown of the integrase cofactor LEDGF/p75 reveals its role in the replication cycle of human immunodeficiency virus J Virol 2006 80 1886 1896 10.1128/JVI.80.4.1886-1896.2006 16439544 PMC1367129

[B65] Zielske SP Stevenson M Modest but reproducible inhibition of human immunodeficiency virus type 1 infection in macrophages following LEDGFp75 silencing J Virol 2006 80 7275 7280 10.1128/JVI.02470-05 16809334 PMC1489053

[B66] Cherepanov P Ambrosio AL Rahman S Ellenberger T Engelman A Structural basis for the recognition between HIV-1 integrase and transcriptional coactivator p75 Proc Natl Acad Sci U S A 2005 102 17308 17313 10.1073/pnas.0506924102 16260736 PMC1297672

[B67] Hare S Shun MC Gupta SS Valkov E Engelman A Cherepanov P A novel co-crystal structure affords the design of gain-of-function lentiviral integrase mutants in the presence of modified PSIP1/LEDGF/p75 PLoS Pathog 2009 5 e1000259 10.1371/journal.ppat.1000259 19132083 PMC2606027

[B68] Petit C Schwartz O Mammano F Oligomerization within virions and subcellular localization of human immunodeficiency virus type 1 integrase J Virol 1999 73 5079 5088 10233971 10.1128/jvi.73.6.5079-5088.1999 PMC112553

[B69] Gabuzda DH Lawrence K Langhoff E Terwilliger E Dorfman T Haseltine WA Sodroski J Role of vif in replication of human immunodeficiency virus type 1 in CD4+ T lymphocytes J Virol 1992 66 6489 6495 1357189 10.1128/jvi.66.11.6489-6495.1992 PMC240141

[B70] Mulder LC Muesing MA Degradation of HIV-1 integrase by the N-end rule pathway J Biol Chem 2000 275 29749 29753 10.1074/jbc.M004670200 10893419

[B71] Mousnier A Kubat N Massias-Simon A Segeral E Rain JC Benarous R Emiliani S Dargemont C von Hippel Lindau binding protein 1-mediated degradation of integrase affects HIV-1 gene expression at a postintegration step Proc Natl Acad Sci U S A 2007 104 13615 13620 10.1073/pnas.0705162104 17698809 PMC1959430

[B72] Llano M Saenz DT Meehan A Wongthida P Peretz M Walker WH Teo W Poeschla EM An essential role for LEDGF/p75 in HIV integration Science 2006 314 461 464 10.1126/science.1132319 16959972

[B73] Li X Koh Y Engelman A Correlation of recombinant integrase activity and functional preintegration complex formation during acute infection by replication-defective integrase mutant human immunodeficiency virus J Virol 2012 86 3861 3879 10.1128/JVI.06386-11 22278243 PMC3302524

[B74] Devroe E Engelman A Silver PA Intracellular transport of human immunodeficiency virus type 1 integrase J Cell Sci 2003 116 4401 4408 10.1242/jcs.00747 13130095

[B75] Llano M Delgado S Vanegas M Poeschla EM Lens epithelium-derived growth factor/p75 prevents proteasomal degradation of HIV-1 integrase J Biol Chem 2004 279 55570 55577 10.1074/jbc.M408508200 15475359

[B76] Tasaki T Mulder LC Iwamatsu A Lee MJ Davydov IV Varshavsky A Muesing M Kwon YT A family of mammalian E3 ubiquitin ligases that contain the UBR box motif and recognize N-degrons Mol Cell Biol 2005 25 7120 7136 10.1128/MCB.25.16.7120-7136.2005 16055722 PMC1190250

[B77] Gallay P Swingler S Aiken C Trono D HIV-1 infection of nondividing cells: C-terminal tyrosine phosphorylation of the viral matrix protein is a key regulator Cell 1995 80 379 388 10.1016/0092-8674(95)90488-3 7859280

[B78] Brussel A Sonigo P Analysis of early human immunodeficiency virus type 1 DNA synthesis by use of a new sensitive assay for quantifying integrated provirus J Virol 2003 77 10119 10124 10.1128/JVI.77.18.10119-10124.2003 12941923 PMC224570

[B79] Thomas JA Ott DE Gorelick RJ Efficiency of human immunodeficiency virus type 1 postentry infection processes: evidence against disproportionate numbers of defective virions J Virol 2007 81 4367 4370 10.1128/JVI.02357-06 17267494 PMC1866140

[B80] Yan N Regalado-Magdos AD Stiggelbout B Lee-Kirsch MA Lieberman J The cytosolic exonuclease TREX1 inhibits the innate immune response to human immunodeficiency virus type 1 Nat Immunol 2010 11 1005 1013 10.1038/ni.1941 20871604 PMC2958248

[B81] Swanstrom R Wills JW Coffin JM Synthesis, assembly, and processing of viral proteins Retroviruses 1997 Plainview, New York: Cold Spring Harbor Laboratory Press 263 334 21433349

[B82] Hare S Gupta SS Valkov E Engelman A Cherepanov P Retroviral intasome assembly and inhibition of DNA strand transfer Nature 2010 464 232 236 10.1038/nature08784 20118915 PMC2837123

[B83] Krishnan L Li X Naraharisetty HL Hare S Cherepanov P Engelman A Structure-based modeling of the functional HIV-1 intasome and its inhibition Proc Natl Acad Sci U S A 2010 107 15910 15915 10.1073/pnas.1002346107 20733078 PMC2936642

[B84] Maroun M Delelis O Coadou G Bader T Segeral E Mbemba G Petit C Sonigo P Rain JC Mouscadet JF Inhibition of early steps of HIV-1 replication by SNF5/Ini1 J Biol Chem 2006 281 22736 22743 10.1074/jbc.M604849200 16772295

[B85] Naldini L Blomer U Gage FH Trono D Verma IM Efficient transfer, integration, and sustained long-term expression of the transgene in adult rat brains injected with a lentiviral vector Proc Natl Acad Sci U S A 1996 93 11382 11388 10.1073/pnas.93.21.11382 8876144 PMC38066

